# Incidence, risk factors, and clinical implications of postoperative blood in or near the resection cavity after glioma surgery

**DOI:** 10.1016/j.bas.2024.102818

**Published:** 2024-04-26

**Authors:** Claes Johnstad, Ingerid Reinertsen, David Bouget, Lisa M. Sagberg, Per S. Strand, Ole Solheim

**Affiliations:** a Department of Neuromedicine and Movement Science, Faculty of Medicine and Health Sciences, Norwegian University of Science and Technology, Trondheim, Norway; b Department of Health Research, SINTEF Digital, Trondheim, Norway; c Department of Circulation and Medical Imaging, Faculty of Medicine and Health Sciences, Norwegian University of Science and Technology, Trondheim, Norway; d Department of Neurosurgery, St. Olav’s Hospital, Trondheim University Hospital, Trondheim, Norway; e Department of Public Health and Nursing, Faculty of Medicine and Health Sciences, Norwegian University of Science and Technology, Trondheim, Norway

**Keywords:** Postoperative hematomas, Surgical complications, Gliomas, Risk factors, Clinical outcomes, Quality of life

## Abstract

**Introduction:**

Postoperative hematomas that require reoperation are a serious, but uncommon complication to glioma surgery. However, smaller blood volumes are frequently observed, but their clinical significance is less known.

**Research question:**

What are the incidence rates, risk factors, and patient-reported outcomes of all measurable blood in or near the resection cavity on postoperative MRI in diffuse glioma patients?

**Material and methods:**

We manually segmented intradural and extradural blood from early postoperative MRI of 292 diffuse glioma resections. Potential associations between blood volume and tumor characteristics, demographics, and perioperative factors were explored using non-parametric methods. The assessed outcomes were generic and disease-specific patient-reported HRQoL.

**Results:**

Out of the 292 MRI scans included, 184 (63%) had intradural blood, and 212 (73%) had extradural blood in or near the resection cavity. The median blood volumes were 0.4 mL and 3.0 mL, respectively. Intradural blood volume was associated with tumor volume, intraoperative blood loss, and EOR. Extradural blood volume was associated with age and tumor volume. Greater intradural blood volume was associated with less headache and cognitive improvement, but not after adjustments for tumor volume.

**Discussion and conclusions:**

Postoperative blood on early postoperative MRI is common. Intradural blood volumes tend to be larger in patients with larger tumors, more intraoperative blood loss, or undergoing subtotal resections. Extradural blood volumes tend to be larger in younger patients with larger tumors. Postoperative blood in or near the resection cavity that does not require reoperation does not seem to affect HRQoL in diffuse glioma patients.

## Highlights

• Hematomas are common on early postoperative MRI after glioma surgery.
• Hematoma volume is associated with intraoperative blood loss, tumor size, and age.
• Small postoperative hematomas do not seem to affect quality of life.

## 1 Introduction

Maximal safe resection is the credo of glioma surgery, attempting to obtain extensive resections that prolong life while minimizing the risk of acquired deficits and complications, including postoperative hematomas. Larger postoperative hematomas that require reoperations are uncommon, with incidence rates ranging from 1 to 3% (Zetterling et al., 2004; Hoover et al., 2013; Ening et al., 2015; Lonjaret et al., 2017; Wang et al., 2019). However, smaller amounts of blood in relation to the surgical approach and resection cavity are frequently observed, but less is known about the potential clinical significance. Postoperative blood in or near the cavities can be observed on early postoperative T1-weighted magnetic resonance imaging (MRI) as hyperintense areas because of the methemoglobin in the blood. A study on gliomas (Ekinci et al., 2003) assessed 50 early post-operative MRI scans and reported blood in or near the resection cavity in 24% of the patients and extra-axial (i.e., epidural, subdural, or subarachnoid) blood in 6%, whereas a smaller study (Forsyth et al., 1997) observed some blood in all seventeen patients. Research on larger postoperative hematomas suggests age (Ening et al., 2015; Wang et al., 2019) and tumor size (Wang et al., 2019) as possible risk factors. Nevertheless, such conclusion might not generalize, and it remains an open question in the case of smaller hematomas. Furthermore, although such hematomas do not cause obvious neurological deficits, there is no current literature on their potential clinical effects. Extravasated blood is potentially neurotoxic, (Stokum et al., 2021) and Fisher grade is a known prognostic factor in subarachnoid hemorrhage and related to cognitive outcome (Ø et al., 2008). In the present study, we sought to assess volumes and incidence rates of all measurable postoperative blood in or near the resection cavity from early postoperative MRI to investigate potential risk factors and potential associations to patient-reported outcomes in diffuse glioma patients.

## 2 Methods and materials

### 2.1 Data

The main source of data for this project is the Central Norway Brain Tumor registry and biobank, which is a population-based registry initiated in 2015 by the St. Olavs Hospital, Trondheim University Hospital (Trondheim, Norway). We accessed the data for research purposes on August 28, 2022. Data missing from this registry were collected from the patient journals, and thus, the first author had brief access to information that could identify individual participants during data collection. In the present study, we analyzed data from 292 diffuse glioma (World Health Organization [WHO] grade 2–4) resections done in 251 patients who underwent surgery between the beginning of December 2015 and the end of December 2022. Histopathology was initially determined according to the WHO classification system used at the time of surgery (i.e., 2007, 2016, or 2021), but we later reclassified the tumors in correspondence with the 2021 WHO classification based on available isocitrate dehydrogenase (IDH) status (available for n = 266) (Louis et al., 2021). We included all patients with available early postoperative (<48 h) T1-weighted MR images without contrast enhancement.

#### 2.1.1 Image assessment

**Fig. 1:**
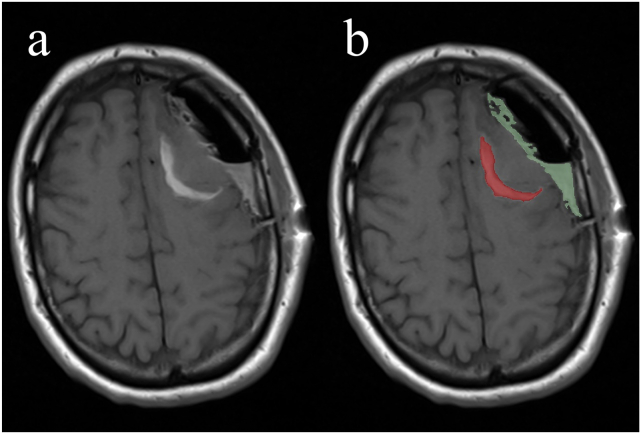
T1-weighted non-enhanced MRI scan exemplifying segmentation of postoperative intradural and extradural blood; a: unedited MRI scan; b: MRI scan after segmentation of blood.

The early postoperative MR images of all patients were described by a neuroradiologist, and measurable volumes of blood in or near resection cavities were segmented by a trained medical student. An experienced neurosurgeon was consulted in cases of doubt. We defined blood as iso-to hyperintense (relative to normal brain tissue) T1 signals without contrast, with hypointense T2 signals in the same area. We manually segmented the blood in 3D Slicer version 5.0.3 using the “Threshold” tool and measured them using the “Labelmap Statistics” tool (Fedorov et al., 2012, Slicer, 2024). We recorded all blood volumes in milliliters with one decimal and categorized them as either extradural or intradural, as shown in Fig. 1. The preoperative tumor volumes were measured by automatic methods developed by our research group (Bouget et al., 2022) and manually corrected by a trained medical student.

#### 2.1.2 Other measures

Postoperative complications were registered from the medical journals at 30 days using the Landriel score and categorized into four groups of severity (Ibañ et al., 2011). We categorized infarctions based on early postoperative diffusion-weighted imaging (DWI) findings, as reported earlier (Strand et al., 2021).

#### 2.1.3 Patient-reported outcomes

Patient-reported health-related quality of life (HRQoL) was scored 1–3 days before surgery and at 30 days after surgery, using the instruments EQ-5D-3L, EORTC QLQ-C30, and QLQ-BN20. EQ-5D-3L is a validated instrument for the assessment of a patient’s generic HRQoL (Rabin et al., 2001). The questionnaire is based on the patient's own evaluation of mobility, self-care, usual activities, pain/discomfort, and anxiety/depression – by which an index value is calculated – and a visual analog scale of their overall health (Rabin et al., 2001). We only assessed the index value and categorized the preoperative to postoperative change as either “improvement”, “worsening”, “no change”, or “missing”. The categories were based on a cutoff value of ±0.13, representing the minimal clinically important difference (Sagberg et al., 2014). The “missing” category comprises all patients whose questionnaires have not been submitted and was included in the analyses as a potential indication of deeper health-related matters. EORTC QLQ-C30 and QLQ-BN20 are questionnaires for measuring disease-specific HRQoL in cancer patients (Fayers and Bottomley, 2002; Taphoorn et al., 2010). We extracted the domains fatigue, headache, and cognitive functioning from these forms and set the cutoff value for worsening and improvement to a change of ±10, respectively (Maringwa et al., 2011).

### 2.2 Statistical analyses

We performed all statistical analyses with RStudio (RStudio, 2023). Q-Q plots and the Shapiro-Wilk test were used to assess the data as non-normally distributed. Hence, we performed non-parametric tests to determine associations between the suspected risk factors and the intradural and extradural blood volumes. We used the Mann-Whitney *U* test for the dichotomous variables and the Kruskal-Wallis test for the polytomous variables. Continuous variables were grouped in quartiles for analyses. Additionally, we conducted two post-hoc multinomial logistic regression analyses to assess the association between intradural blood volume and change in cognitive functioning and headache, respectively, when adjusting for tumor volume. Changes in cognitive functioning and headache were set as the respective response variables, whereas tumor volume, blood volume, and their interaction were set as predictor variables. We conducted another post-hoc Kruskal-Wallis analysis on the association between location of the tumors and postoperative blood volumes, using Montreal Neurological Institute (MNI) brain atlas to define lobar borders (Fonov et al., 2009). The location of each tumor was computed automatically and defined as the atlas lobe with which most of the tumor segmentation overlapped (Bouget et al., 2022). We defined statistical significance as p < 0.05. Significant p-values were also adjusted for multiple testing according to the Holm-Bonferroni method to reduce the risk of type I errors. The results are presented as the median with the first and third quartile (Q1-Q3) or as p-value of the relevant analysis.

### 2.3 Ethics

All parts of this project were conducted in accordance with the Declaration of Helsinki. The project has been approved by the Regional Committee for Medical and Health research ethics (REK) in Norway (REK-reference 2019/510). Active, written, informed consent was obtained from all patients.

## 3 Results

**Fig. 2:**
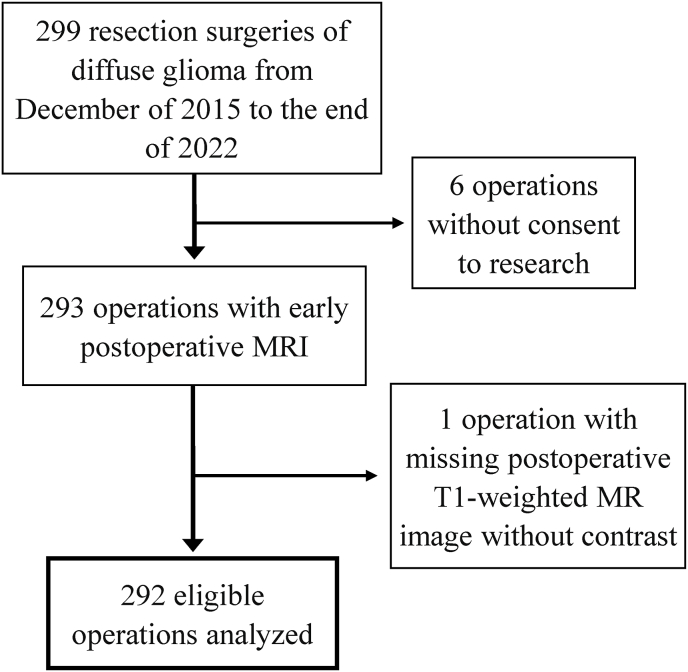
Flowchart of inclusion process.

Fig. 2 shows a flowchart of the patient inclusion. As seen, 299 glioma resections were performed in the study period. We excluded six patients due to a lack of informed consent to research and one because of a missing non-enhanced T1-weighted MRI scan. In total, we included and analyzed data from 292 glioma resections of 251 patients.

### 3.1 Incidence rates

**Fig. 3:**
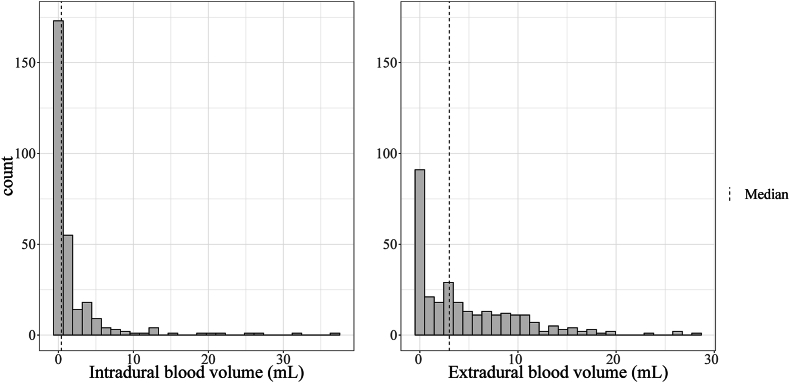
Distribution of blood volumes in or near the resection cavity; left: distribution of intradural blood volumes; right: distribution of extradural blood volumes.

After 184 (63%) of the operations, a measurable intradural blood volume of 0.1 mL or more could be observed on early postoperative MRI, while a measurable extradural blood volume was seen after 212 (73%) of the operations. The median volumes of intradural and extradural blood components were 0.4 and 3.0 mL, respectively. Fig. 3 shows the distribution of intradural and extradural blood volumes. One patient (0.3%) was reoperated for a late occurring larger intradural hematoma 5 days after surgery but only had 0.1 mL of intradural blood on the early postoperative MRI. Three patients (1.0%) were reoperated for late occurring, symptomatic postoperative extradural hematomas between 5 and 19 days postoperatively. These extradural hematomas measured 8.4, 3.1, and 6.0 mL, respectively, at the time of early postoperative MRI, but grew subsequently.

### 3.2 Potential risk factors

Table 1 shows the median intradural and Table 2 shows the median extradural blood volumes for explored potential risk factors. We observed a positive significant association between intradural blood volumes and preoperative tumor volumes, more intraoperative blood loss, and subtotal extents of resection. There was also a significant association between extradural blood volume and both lower age and greater tumor size, respectively.

**Table 1:** Explored potential risk factors for measurable intradural blood volume. mL: milliliters; Q1: first quartile; Q3: third quartile; WHO: World Health Organization; DWI: diffusion-weighted imaging.

	Median mL intradural blood volume (Q_1_-Q_3_)	p-value	Adjusted p-value
Histopathology		0.091	
WHO grade 2 (n = 48)	0.2 (0.0–0.7)		
WHO grade 3 (n = 40)	0.2 (0.0–1.1)		
WHO grade 4 (n = 199)	0.5 (0.0–2.3)		
Sex		0.073	
Male	0.2 (0.0–1.2)		
Female	0.5 (0.0–2.3)		
Age quartiles		0.97	
1. (20–43)	0.3 (0.0–1.3)		
2. (44–57)	0.3 (0.0–1.4)		
3. (57–65)	0.5 (0.0–1.2)		
4. (65–82)	0.4 (0.0–2.5)		
Preoperative tumor volume quartiles (range in mL)		**0.00039**	**0.011**
1. (0.16–7.32)	0.1 (0.0–0.8)		
2. (7.49–22.9)	0.3 (0.0–1.2)		
3. (23.6–47.9)	0.5 (0.0–2.3)		
4. (48.5–285.0)	0.9 (0.1–3.0)		
Intraoperative blood loss quartiles (range in mL)		**0.0011**	**0.031**
1. (0–60)	0.2 (0.0–1.0)		
2. (60–120)	0.4 (0.0–2.3)		
3. (120–250)	0.1 (0.0–0.8)		
4. (250–1000)	0.8 (0.1–4.4)		
Preoperative systolic blood pressure quartiles (range)		0.93	
1. (75–113)	0.5 (0.0–1.6)		
2. (113–128)	0.3 (0.0–1.4)		
3. (128–142)	0.4 (0.0–1.3)		
4. (142–195)	0.4 (0.0–1.6)		
Postoperative systolic blood pressure quartiles (range)		0.15	
1. (81–120)	0.4 (0.0–2.4)		
2. (120–132)	0.1 (0.0–0.8)		
3. (132–144)	0.5 (0.0–2.0)		
4. (144–183)	0.5 (0.0–1.7)		
Complications		0.25	
No registered complications (n = 215)	0.5 (0.0–1.7)		
Grade 1 complications (n = 58)	0.2 (0.0–1.5)		
Grade 2 complications (n = 14)	0.1 (0.0–0.2)		
Grade 3 complications (n = 5)	0.0 (0.0–0.5)		
Grade 4 complications (n = 1)	4.5 (4.5–4.5)		
Postoperative infarction on DWI		0.13	
No infarction (n = 171)	0.3 (0.0–1.7)		
Rim only (n = 52)	0.1 (0.0–1.2)		
Sector infarction ± rim (n = 62)	0.5 (0.0–2.5)		
Extent of resection		**0.037**	0.85
Partial resection quartiles (range)			
1. (2.7%–64.8%)	0.3 (0.0–1.7)		
2. (65.6%–87.6%)	0.8 (0.18–1.7)		
3. (87.9%–96.3%)	0.8 (0.0–2.4)		
4. (96.4%–99.9%)	0.7 (0.0–2.6)		
Gross total resection	0.1 (0.0–1.0)		
Tumor location		0.23	
Frontal lobe (n = 145)	0.2 (0.0–1.2)		
Occipital lobe (n = 8)	0.7 (0.4–0.9)		
Parietal lobe (n = 46)	0.4 (0.0–2.2)		
Temporal lobe (n = 92)	0.6 (0.0–2.1)		
Thalamus (n = 1)	0.2 (0.2–0.2)

**Table 2:** Explored potential risk factors for measurable extradural blood volume. mL: milliliters; Q1: first quartile; Q3: third quartile; WHO: World Health Organization.

	Median mL extradural blood volume (Q_1_-Q_3_)	p-value	Adjusted p-value
Sex		0.16	
Male	2.7 (0.0–7.2)		
Female	3.4 (0.0–8.4)		
Age quartiles		**0.0066**	0.16
1. (20–43)	4.8 (1.7–8.4)		
2. (44–57)	3.6 (0.3–8.5)		
3. (57–65)	1.5 (0.0–6.3)		
4. (65–82)	2.0 (0.0–6.0)		
Preoperative tumor volume – quartiles (range)		**0.0023**	0.060
1. (0.16–7.32)	2.8 (0.0–7.2)		
2. (7.49–22.9)	2.0 (0.0–6.4)		
3. (23.6–47.9)	2.5 (0.0–6.2)		
4. (48.5–285.0)	5.4 (2.0–10.0)		
Preoperative systolic blood pressure quartiles (range)		0.38	
1. (75–113)	3.6 (0.5–7.5)		
2. (113–128)	3.1 (0.8–7.8)		
3. (128–142)	2.8 (0.0–7.75)		
4. (142–195)	1.7 (0.0–6.3)		
Postoperative systolic blood pressure quartiles (range)		0.075	
1. (81–120)	3.3 (0.6–8.7)		
2. (120–132)	3.6 (0.5–9.0)		
3. (132–144)	2.6 (0.0–7.6)		
4. (144–183)	1.9 (0.0–5.8)		
Complications		0.81	
No registered complications (n = 215)	2.8 (0.0–7.8)		
Grade 1 complications (n = 58)	3.4 (0.0–7.2)		
Grade 2 complications (n = 14)	3.2 (1.3–8.1)		
Grade 3 complications (n = 5)	0.9 (0.0–6.0)		
Grade 4 complications (n = 1)	5.7 (5.7–5.7)		
Tumor location		0.33	
Frontal lobe (n = 145)	2.8 (0.0–7.1)		
Occipital lobe (n = 8)	0.5 (0.0–3.0)		
Parietal lobe (n = 46)	3.0 (0.0–7.7)		
Temporal lobe (n = 92)	3.6 (0.3–8.9)		
Thalamus (n = 1)	2.3 (2.3–2.3)		

### 3.3 Patient-reported outcomes

**Fig. 4:**
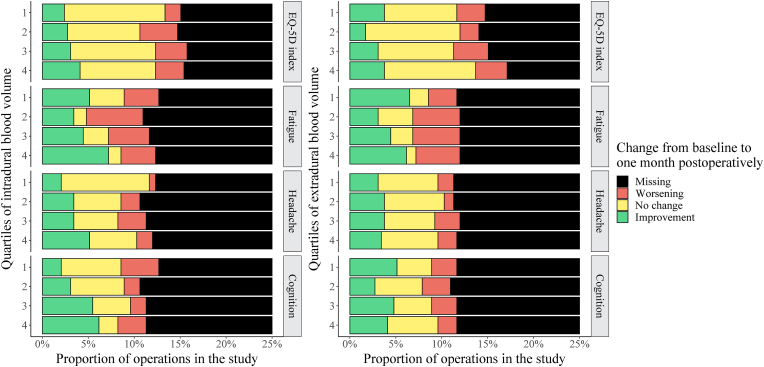
Change in patient-reported outcomes from preoperative assessments to 30 days postoperatively grouped by measured intradural (left) and extradural (right) blood volumes; Quartile 1: smallest blood volumes; Quartile 4: largest blood volumes.

We retrieved and compared available preoperative and postoperative questionnaires on generic overall HRQoL, fatigue, headache, and cognitive functioning for 178, 139, 135, and 134 of the operations, respectively. Fig. 4 shows the distribution of whether the patients reported worsening, improvement, or no change from preoperative measurements to one month postoperatively, for each intra- and extradural blood volume quartile. We observed statistical associations between intradural blood volume and less headache (p = 0.037, adjusted p = 0.85) as well as improvement of cognitive functioning (p = 0.0031, adjusted p = 0.077). We found no significant associations between intradural blood volumes and changes in neither EQ-5D-3L index values (p = 0.27) nor fatigue (p = 0.17). Neither were there any significant associations between measured extradural blood volumes and changes in EQ-5D-3L index values (p = 0.58), reported cognitive functioning (p = 0.51), fatigue (p = 0.23), nor headache (p = 0.73). A post-hoc analysis showed an association between preoperative tumor volume and improvement of both headache (p = 0.0017, adjusted p = 0.046) and cognitive functioning (p < 0.0001, adjusted p < 0.0001), respectively. An additional multinomial logistic regression analysis showed no association between intradural blood volume and risk of neither less headache (p = 0.65) nor cognitive improvement (p = 0.92) when adjusting for tumor volume.

## 4 Discussion

In this retrospective, population-based cohort study, we observed measurable intradural and extradural blood volumes in early postoperative MRIs after 63% and 72% of glioma resection surgeries, with a median volume of 0.4 and 3.0 mL, respectively. Larger preoperative tumor volume, greater intraoperative blood loss, and subtotal extents of resection were associated with larger intradural blood volumes. Lower age and larger tumors were associated with larger extradural blood volumes, although not statistically significant after p-value adjustments. Interestingly, there were no associations to perioperative blood pressures, tumor location, or WHO tumor grade. The clinical consequences of the frequent, but mostly small, blood volumes are not clear, as we could not observe associations to registered complications, nor dose-response associations to overall HRQoL or fatigue. Although patient-reported improvement regarding headache and cognitive functioning after glioma surgery was associated with larger intradural blood volumes, tumor volume is likely a confounder.

Some previous studies have been conducted on the incidence and potential risk factors of postoperative hematomas (Zetterling et al., 2004; Hoover et al., 2013; Ening et al., 2015; Lonjaret et al., 2017; Wang et al., 2019). However, they only studied larger hematomas, requiring surgical evacuation, whose risk factors may not translate to all blood volumes in or near the resection cavity. In addition, most previous studies included all craniotomies or operations of all intracranial tumors, instead of glioma resections specifically. Thus, their results may not be fully comparable to ours. Nonetheless, tumor size was identified as a potential risk factor in our analyses, which is in line with other studies on postoperative hematomas. In 2019, a retrospective cohort study of 2259 intracranial tumor resections found preoperative tumor diameter to be a risk factor for postoperative hematomas (Wang et al., 2019). When resecting larger gliomas, the increased resection cavity space can favor potential intradural hematomas. Furthermore, the resection surface from which bleeding may occur is greater, which could explain these findings. Our results also showed a similar trend for extradural blood volumes. After a large tumor resection, the dura may sag towards the cavity after closure, opening a space for an extradural hematoma to build up. Additionally, larger tumors often require larger craniotomies, perhaps also explaining this finding.

A Swedish study showed a significant association between the risk of postoperative hematoma and intraoperative blood loss, (Zetterling et al., 2004) which is also supported by our findings for intradural blood in or near the resection cavity. A lack of coagulation factors and platelets in the patients' blood, causing an increased bleeding tendency, might be the explanation. Additionally, more intraoperative bleeding leads to a cluttered surgical cavity, hindering the achievement of successful hemostasis. From previous reported findings associating intraoperative blood loss with postoperative infarctions, (Strand et al., 2021) we hypothesized that infarctions were similarly associated with measured blood volumes in or near the cavity, which was however not supported by our data. Furthermore, we expected that a greater extent of resection would be a protective factor for bleeding volume, as tumor tissue tends to bleed more than normal brain parenchyma (Ostrowski et al., 2022). As expected, operations with gross total resection typically had smaller intradural blood volumes than those with subtotal resection. Yet, operations with the lowest extent of resection also had smaller measurable blood volumes than those with greater subtotal resection. Smaller resection cavities in these patients, allowing for less space for potential bleeding, might be a reasonable rationalization. This association was, however, not significant after the Holm-Bonferroni adjustment.

Blood pressure is thoroughly monitored perioperatively. While perioperative hypertension has been linked to postoperative hematomas in need of reoperations in some, (Basali et al., 2000; Seifman et al., 2011) but not all (Wang et al., 2019) studies, we found no association to radiologically measured blood volumes in the present study. Contrary to our expectations, the median extradural blood volume decreased with increasing postoperative systolic blood pressure. However, this trend was not statistically significant (p = 0.075), and age could perhaps be a confounder. Some studies have identified an association between higher age and risk of postoperative hematomas, (Wang et al., 2019; Palmer et al., 1994; Lassen et al., 2011) but no association between age and intradural blood volumes was corroborated in our study. Conversely, we found that younger patients tended to have larger extradural blood volumes, which could potentially be explained by the looser attachment between the dura and overlying bone adjacent to the cavity. We also expected the WHO grade of the tumors to be associated with intradural blood volumes, as more malignant gliomas tend to exhibit greater neovascularization and infiltration of normal brain tissue (Ahir et al., 2020; Leon et al., 1996). However, WHO grade was not associated with intradural blood volumes in our study. Additionally, the post-hoc analysis on tumor location showed no association between the lobar location of the tumor and post-operative blood volume in or near the resection cavity, as supported by other studies on hematomas (Ening et al., 2015; Wang et al., 2019). However, categorizing multilobar tumors into singular lobes based on proportional overlap, simplifies the data and may be a weakness.

There is no agreed-upon definition of a postoperative hematoma, and indications for reoperation may also vary. Most, if not all brain operations are associated with minute, but often unmeasurable volumes of blood in or near the surgical field. In the present study, we assessed all measurable blood volumes and hypothesized that the frequent smaller postoperative blood volumes in or near the surgical field still could have clinical consequences for the patients, even though they do not require reoperations. Keeping in mind that missing patient-reported outcomes may not be missing at random, we included this group in the analyses. There was no clear dose-response association between measured blood volumes and overall generic HRQoL or fatigue. However, we found that patient-reported improvement of cognitive functioning and less headache after glioma surgery was associated with larger intradural blood volumes. This could suggest that minute hematomas not requiring reoperations may still affect patients' quality of life or subjective function. Even so, the observed trend is likely confounded by tumor size, which has been associated with both larger intradural hematoma volumes and cognitive improvement after surgery (Schei et al., 2022). However, other studies on cognitive change in glioma patients report no significant association with tumor volume (Barzilai et al., 2019; Zarino et al., 2021; Noll et al., 2016). Thus, we performed a post-hoc analysis, in which we observed an association between larger tumor volumes and improvement of both headache and cognitive functioning, which suggests that tumor volume may have functioned as a confounder in the initial analyses. Further, we conducted a multinomial logistic regression analysis to adjust for tumor volume, in which we found no associations between intradural blood volumes and increased risk of neither improvement of cognitive functioning nor less headache. Consequently, hematomas or blood volumes that do not require reoperation do not appear to significantly affect the patients' HRQoL.

Explorative studies like this are not meant to guide clinical practice alone. Nevertheless, our results support the current assumption that minor blood volumes in or near the resection cavity do not significantly affect the patients. Consequently, there appears to be no need for targeted monitoring of postoperative blood volume or treatment of blood in or near the resection cavity that does not cause obvious neurological deficits during the postoperative follow-up of patients with diffuse gliomas. Furthermore, as postoperative blood in or near the resection cavity does not seem to harm the patients, any risk factors for such should not affect the preoperative risk assessment or surgical procedure. We acknowledge that risk factors for small amounts of blood in or near the resection cavity may not be shared with those of large, clinically significant hematomas.

The greatest strengths of this study are the sample size, the population-based patient selection that minimizes referral bias, and the consideration of all measurable blood volumes, regardless of size. Additionally, we have segmented all the identifiable blood on early postoperative MRI and used the volumes as a continuous variable, instead of dichotomizing them as “yes” or “no”. However, diagnosing or measuring hematomas from postoperative MRI is difficult, as blood signal changes over time due to the oxidation of hemoglobin, (Maizlin et al., 2009) and liquid blood, bloody CSF, and bloody saline exhibit different signals than coagulated blood. MR images were taken 12–48 h after surgery, but the dynamic change in the mix of oxyhemoglobin, deoxyhemoglobin, and methemoglobin affects both T1 and T2 signals over time (Bradley, 1993). It can therefore be difficult to determine an exact threshold for which signal intensities to define as hematomas or blood on different MRI sequences. Previous studies have focused on hematomas that require surgical evacuation only and used this as a dichotomous outcome variable in their analyses, (Zetterling et al., 2004; Hoover et al., 2013; Ening et al., 2015; Lonjaret et al., 2017; Wang et al., 2019) ensuring the clinical relevance. To our knowledge, this is the first study to assess the full range of postoperative blood as a volumetric measure in unselected diffuse glioma patients undergoing resection. Although most of the blood volumes measured in this study were obviously too small to be clinically significant, the continuous scale was necessary to avoid disregarding blood volumes below any cutoff value, which there is no current literature to justify. A continuous volume variable strengthens the statistical analyses and provides grounds for the current assumptions in clinical practice that minor hematomas cause no significant harm to the patients and can be disregarded.

However, manual segmentations of the blood volumes also introduce limitations. Although the segmentations were performed by a trained medical student with the help of an experienced neurosurgeon, we did not perform any inter-rater reliability checks to assess the accuracy of the annotations, which is a possible weakness in the measurements. However, there is currently no gold standard or verified way of measuring postoperative blood volume in or near the resection cavity. Thus, although somewhat subjective, we considered manual segmentation the best option. Furthermore, very small blood segments could more likely be false positives, the smallest of which may be enhanced by for example hemostatic agents used intraoperatively (e.g. Surgicel®). It is difficult to separate the coagulants from postoperative blood, and they cannot be subtracted using MRI scans only, thus introducing a potential weakness. However, the coagulants unlikely affected the conclusion in our study, as these volumes were very small. Another factor that could impact the blood volumes is the use of valproic acid; seizures are a common presenting symptom of gliomas in adults, (Posti et al., 2015) and treatment with valproic acid may cause coagulopathy in these patients, (Kumar et al., 2019) potentially resulting in confounding. Causal inference is difficult to establish from observational data, and potential confounders should always be considered. As a single-center study, the external validity is also reduced due to potential variations in clinical practice between hospitals and countries. Still, our center serves exclusively in a defined geographical region with population-based referral, and the cases included are therefore unselected. Although we were unable to detect any clear consequences for HRQoL, we cannot exclude that other HRQoL instruments or neuropsychological tests could be more sensitive in this setting.

## 5 Conclusion

We observed radiologically measurable intradural and extradural blood in or near the surgical cavity on early postoperative MRI after 63% and 73% of the glioma resections, respectively. However, both measured intradural and extradural blood volumes were usually small with median volumes of 0.4 and 3.0 mL, respectively. Tumor volume and intraoperative blood loss were associated with larger intradural blood volumes. Extradural blood volumes were larger in younger patients and in patients with bigger tumors. Patients with larger intradural blood volumes more often reported less headache and improved cognitive functioning compared to preoperatively. However, tumor size is most likely a confounder, and consequently, the frequent minor postoperative hematomas or blood volumes do not appear to affect health-related quality of life in diffuse glioma patients.

## Funding

Open access funding provided by 10.13039/100009123NTNU 10.13039/100009123Norwegian University of Science and Technology (including St. Olavs Hospital - Trondheim University Hospital).

## Ethical approval

The project was ethically approved by the Regional Committee for Medical and Health research ethics (REK) in Norway (REK-reference 2019/510).

## Patient consent

All patients gave informed, written consent to research before the start of the inclusion process for this study. All parts of the study were conducted in accordance with the Declaration of Helsinki.

## Declaration of competing interest

The authors declare that they have no conflict of interest.
